# Supplementary material for: Knowledge and Awareness of HPV, the HPV Vaccine and Cancer-Related HPV Types among Indigenous Australians
Source: Int J Environ Res Public Health. 2024 Mar 6;21(3):307. doi: 10.3390/ijerph21030307 (PMC10970159; doi:10.3390/ijerph21030307)
Supplement: Supplementary file 1 [file ijerph-21-00307-s001.zip › ijerph-2781044-supplementary.pdf]

Knowledge and Awareness of HPV, the HPV Vaccine and HPV-related Cancers among Indigenous Australians

Lucy Lockwood [1], Xiangqun Ju [1]*, Sneha Sethi[1], Joanne Hedges[1], Lisa Jamieson [1,]

[1]Australian Research Centre for Population Oral Health (ARCPOH), Adelaide Dental School, University of Adelaide, Adelaide, Australia; lucy.lockwood@student.adelaide.edu.au; xiangqun.ju@adelaide.edu.au; sneha.sethi@adelaide.edu.au; joanne.hedges@adelaide.edu.au; lisa.jamieson@adeelaide.edu.au

*Correspondence: xiangqun.ju@adelaide.edu.au; Tel.: (61 8 83137352)

Supplementary Tables

Table S1: 10-item Human Papilloma Virus (HPV) Knowledge Tool (12-month follow up survey – Section A)

A. PLEASE LOOK AT THESE STATEMENTS AND SAY WHETHER YOU THINK EACH ONE IS TRUE OR FALSE			
A1. HPV is very rare	True ☐$_1$	False ☐$_2$	Don't know ☐$_3$
A2. HPV always has visible signs or symptoms	True ☐$_1$	False ☐$_2$	Don't know ☐$_3$
A3. HPV can be passed on by genital skin-to-skin contact	True ☐$_1$	False ☐$_2$	Don't know ☐$_3$
A4. There are many types of HPV	True ☐$_1$	False ☐$_2$	Don't know ☐$_3$
A5. HPV can be passed on during sexual intercourse	True ☐$_1$	False ☐$_2$	Don't know ☐$_3$
A6. Men cannot get HPV	True ☐$_1$	False ☐$_2$	Don't know ☐$_3$
A7. HPV usually doesn't need any treatment	True ☐$_1$	False ☐$_2$	Don't know ☐$_3$
A8. Most sexually active people will get HPV at some point in their lives	True ☐$_1$	False ☐$_2$	Don't know ☐$_3$
A9. A person could have HPV for many years without knowing it	True ☐$_1$	False ☐$_2$	Don't know ☐$_3$
A10. HPV can cause cancer in men	True ☐$_1$	False ☐$_2$	Don't know ☐$_3$

Where correct answers are; A1 False, A2 False, A3 True, A4 True, A5 True, A6 False, A7 True, A8 True, A9 True, A10 True.

Table S2: Baseline and 12-moth follow up survey on HPV vaccine status.

1. Baseline questionnaire-Section F
   F2: Have you ever received a vaccination for HPV? Option: 1) Yes, 2) No or 3) Don't know.
2. 12-moth follow up survey - Section E

E. THESE QUESTIONS ARE ABOUT THE HPV VACCINATION: *BOTH BOYS AND GIRLS CAN RECEIVE THE HPV VACCINE*

E1. Which of the following statements best describes whether you have had the HPV vaccine (also called the human papillomavirus vaccine, cervical cancer vaccine, Gardasil or Cervarix vaccine)?

Please select only ONE option

Yes, I had 1 dose of the HPV vaccine	Yes, I had 2 doses of the HPV vaccine	Yes, I had 3 doses of the HPV vaccine	Yes I have but I am not sure how many doses I received	No, I was offered the HPV vaccine, but I did not have it	No, I have never been offered the HPV vaccine	I don't know
☐1	☐2	☐3	☐4	☐5 *(Go to F1)*	☐6 *(Go to F1)*	☐7 *(Go to F1)*

Any comments?

E2. Where did you receive your HPV vaccine (also called the human papillomavirus vaccine, cervical cancer vaccine, Gardasil or Cervarix vaccine)?

Please select only ONE option for each dose

1st dose

School	General practitioner/practice nurse	Family Planning Clinic	Specialist doctor	Can't remember	Other:
☐1	☐2	☐3	☐4	☐5	☐6 Please specify: ______

2nd dose

School	General practitioner/practice nurse	Family Planning Clinic	Specialist doctor	Can't remember	Other:
☐1	☐2	☐3	☐4	☐5	☐6 Please specify: ______

3rd dose

School	General practitioner/practice nurse	Family Planning Clinic	Specialist doctor	Can't remember	Other:
☐1	☐2	☐3	☐4	☐5	☐6 Please specify: ______

E3. Which years were you vaccinated with the HPV vaccine (also called human papillomavirus vaccine, cervical cancer vaccine, Gardasil or Cervarix vaccine)? (e.g. 2002)

The HPV vaccine first became available free for girls in year 7 in Australia from 2007 and this is ongoing. It was also available free of charge to women aged up to 26 years from 2007 until the end of 2009. The vaccine was available for boys from 2013.

1st dose							Can't remember ☐1
2nd dose							Can't remember ☐1
3rd dose							Can't remember ☐1

Table S3: 12-moth follow up survey - Section C

C7. Before today, had you ever heard of HPV (human papillomavirus)?				Yes ☐1	No ☐2	Not sure ☐3
C8. Do you think your knowledge about HPV is:	Never heard of HPV ☐1	Very poor ☐2	Poor ☐3	Fair ☐4	Good ☐5	Very good ☐6
C9. Do you have any unanswered questions about HPV?		Yes ☐1	Please state: ______			No ☐2

Table S4: 10 items HPV-KT responses

	HPV-KT number of each response									
Question	1	2	3	4	5	6	7	8	9	10
True/1	133	120	**241***	**260***	**355***	62	**72***	**232***	**382***	**259***
False/2	**167***	**168***	70	28	32	**357***	325	92	12	29
Don't know/3	444	457	431	457	357	328	346	420	351	455
Correct %	22.4	22.6	32.5	34.9	47.7	47.8	9.7	31.2	51.3	34.9

Bold* indicates correct answer
